# Surgical management of bilateral hip fractures in a patient with fibrodysplasia ossificans progressiva treated with the RAR-γ agonist palovarotene: a case report

**DOI:** 10.1186/s12891-020-03240-2

**Published:** 2020-04-03

**Authors:** Sukhmani Singh, Joseph Kidane, Kelly L. Wentworth, Daria Motamedi, Saam Morshed, Andrew E. Schober, Edward C. Hsiao

**Affiliations:** 1 Division of Endocrinology and Metabolism, Department of Medicine, the Institute for Human Genetics; and the Program in Craniofacial Biology - University of California, San Francisco, 513 Parnassus Ave., HSE901, San Francisco, CA 94143-0794 USA; 2 Department of Medicine, University of California, San Francisco, 533 Parnassus Ave, San Francisco, CA 94143 USA; 3 Department of Radiology, University of California, San Francisco, 505 Parnassus Ave, San Francisco, CA 94143 USA; 4 Department of Orthopedic Surgery, University of California, San Francisco, and the Orthopedic Trauma Institute, 2550 23rd Street, Building 9, 2nd Floor, San Francisco, CA 94110 USA; 5 Department of Anesthesiology, University of California, San Francisco, 521 Parnassus Ave, San Francisco, CA 94131 USA

**Keywords:** Fibrodysplasia ossificans progressiva (FOP), Palovarotene, Retinoic acid receptor (RAR) agonists, Heterotopic ossification (HO), Prophylaxis, Hip fracture, Repair surgery, Case report

## Abstract

**Background:**

Fibrodysplasia ossificans progressiva (FOP) is an ultra-rare disorder marked by painful, recurrent flare-ups and heterotopic ossification (HO) in soft and connective tissues, which can be idiopathic or provoked by trauma, illness, inflammation, or surgery. There are currently no effective treatments for FOP, or for patients with FOP who must undergo surgery. Palovarotene, an investigational retinoic acid receptor-γ agonist, offers a potential avenue to prevent HO formation.

**Case presentation:**

The patient is a 32 year-old male, who at age 29 enrolled in a study evaluating palovarotene to prevent HO formation in FOP. One year after starting palovarotene, he fell resulting in a left intertrochanteric fracture. He underwent intramedullary nailing of the femur shaft with screw placement at the distal femur. After surgery, he received palovarotene at 20 mg/day for 4 weeks, then 10 mg/day for 8 weeks. Imaging 12 weeks after surgery showed new bridging HO at the site of intramedullary rod insertion and distal screw.

Nine months after the left hip fracture, the patient had a second fall resulting in a subdural hematoma, left parietal bone fracture, and right intertrochanteric fracture. He underwent intramedullary nailing of the right hip, in a modified procedure which did not require distal screw placement. Palovarotene 20 mg/day was started at fracture occurrence and continued for 4 weeks, then reduced to 10 mg/day for 8 weeks. HO also formed near the insertion site of the intramedullary rod. No HO developed at the right distal intramedullary rod. After each fracture, the patient had prolonged recurrent flare-ups around the hips.

**Conclusion:**

Surgery is only rarely considered in FOP due to the high risks of procedural complications and potential for inducing HO. This case emphasizes the risks of increased flare activity and HO formation from injury and surgery in patients with FOP. The efficacy of HO prevention by palovarotene could not be assessed; however, our observation that palovarotene can be administered in an individual with FOP following surgery with no negative impact on clinical fracture healing, osteointegration, or skin healing will help facilitate future trials testing the role of palovarotene as a therapy for HO.

## Background

Heterotopic ossification (HO) is a severe condition of bone formation in an inappropriate anatomic location, often triggered by trauma or other inflammatory processes [1]. One genetic condition that leads to massive and progressive HO is fibrodysplasia ossificans progressiva (FOP), a rare debilitating disease resulting from mutations in the Activin A Receptor Type 1 (ACVR1)/Activin-like Kinase 2 (ALK2) gene encoding for a type 1 membrane receptor for bone morphogenetic proteins (BMPs) [2]. This mutation leads to over-activation of downstream SMAD pathways which trigger the formation of excessive heterotopic endochondral bone [3].

FOP may be diagnosed in childhood by findings of malformations of the great toes combined with unusual indurated masses that eventually form bone. Patients with FOP develop painful soft tissue swellings (“flare-ups”), which can be precipitated by trauma, injury, illness, or other inflammatory processes. These flare-ups can progress to HO and progressive loss of mobility [4, 5]. For this reason, any type of injury, including iatrogenic injury from medical or surgical procedures, must be minimized. Current therapies to reduce disease flare-ups are limited, with steroids and non-steroidal anti-inflammatory agents (NSAIDs) currently being used for symptom management though they may not alter disease course [5].

Palovarotene is a retinoic acid receptor (RAR) γ agonist that can reduce heterotopic bone formation in mouse models of FOP [6]. Studies in mice have also suggested that retinoids may be associated with a transient delay in fracture healing which may have implications for patients on treatment [7]. Ongoing clinical trials suggest that palovarotene may be administered as episodic treatments for flare-ups (NCT02190747, NCT03312634). Here we present a case study showing that routine fracture healing after surgical repair of two hip fractures in an individual with FOP on palovarotene treatment showed no clinically significant delay in fracture healing and that the medication was not associated with any unexpected adverse events.

## Case presentation

The patient is a 32 year-old male at the time of this case report with a history of asthma, Gilbert’s disease, and hypertension who was diagnosed with FOP at age 9 after presenting with a limp. At presentation, he had calcification in his left sartorius muscle on imaging. He subsequently had FOP bone formation flare-ups with increasing frequency leading to reduced mobility, though he remained independent for activities of daily living and ambulated without assistive devices. The major joints affected were his spine, jaw, shoulders, right hip, and bilateral ankles. From his teens to his mid-twenties flare-ups were treated with a prednisone taper following standard-of-care guidelines [8]. Between ages 13 to 28 years he also received 4-week courses of thalidomide for flare-ups as part of a clinical study, for its anti-angiogenic effect, but without major clinical changes in his disease course [9]. He received his first dose of zoledronic acid, in an effort to reduce flare-ups through possible anti-inflammatory effects [10], at approximately age 19 years, then received 8 doses between the ages of 25 to 29 years and yearly thereafter.

**Fig. 1:**
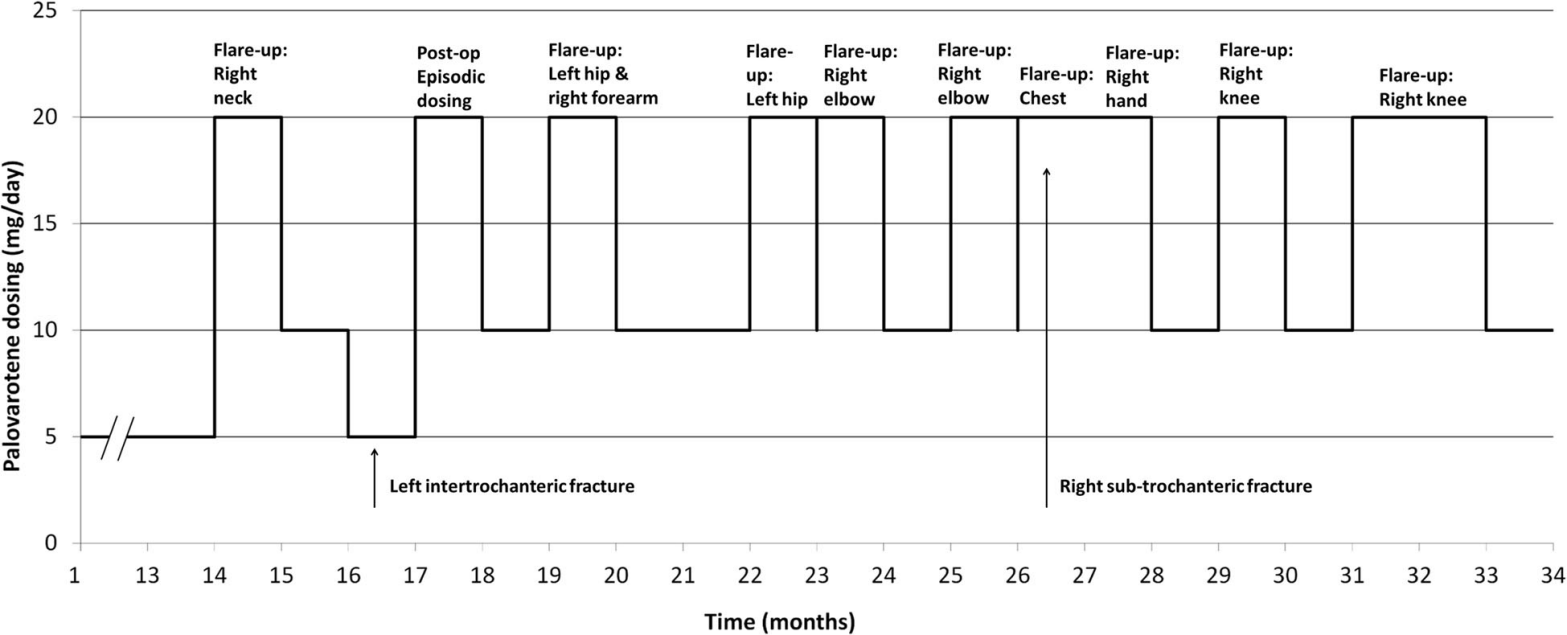
Timeline of palovarotene dosing in the open label phase of the PVO-1A-202B trial. Flare-up dosing, flare site, and fracture events are noted

At age 29 years, he enrolled in the phase 2 randomized control trial of palovarotene for the treatment of preosseous flare-ups in FOP (NCT02190747). He was having approximately 4 flares per year at the time of enrollment. He completed 6 weeks on the study protocol and subsequently enrolled in an open-label extension trial for the use of palovarotene (episodic dosing of 20 mg/day for 4 weeks, then 10 mg/day for 8 weeks) for flare-ups (NCT02279095). He then transitioned to a chronic treatment arm with palovarotene (5 mg/day) along with the episodic treatment with palovarotene for flare-ups. In addition to palovarotene, he received prednisone 110 mg/day for 4 days followed by a taper for his flare-up treatments. His treatment course and flare-up activity in the open-label extension trial is depicted in Fig. 1. During this time, the patient received topical prophylaxis with Aquaphor barrier ointment, triamcinolone 0.1% for retinoid skin reactions such as rash and dry skin, and artificial tears, for managing the most common potential mucocutaneous side effects of palovarotene.

**Fig. 2:**
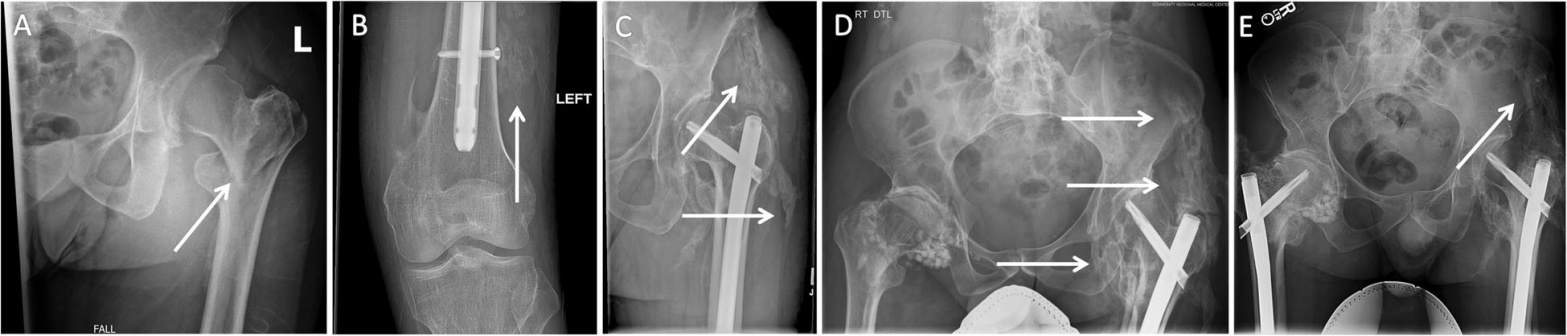
X-Ray imaging of the left hip: (**a**) X-Ray of the left pelvis at the time of the subtrochanteric fracture [arrow]. (**b**) X-Ray of the knee 4 months after surgery with HO at the site of distal screw insertion [arrow]. (**c**) Brooker class D HO at the rod insertion site 4 months after surgery [arrows] and after 9 months (**d**). (**e**) X-ray showing matured HO formation at 12 months [arrow]

One year after starting chronic palovarotene treatment, the patient had a fall resulting in a left inter-trochanteric fracture (Fig. 2). He was on palovarotene 5 mg/day, and had just completed a palovarotene/prednisone treatment cycle two weeks prior. Palovarotene 5 mg/day treatment was interrupted for one day after the fall then resumed at 5 mg/day until his surgery. Immediately after the fall, he was started on high-dose prednisone (110 mg/day) to reduce inflammation at the surgical site [5]. High-dose prednisone was continued until 3 days post-op and then tapered by 10 mg/day until completely stopped. To minimize the risk of flare-ups, intravenous (IV) treatments were discontinued as early as possible and aspirin (81 mg twice daily) was used for deep vein thrombosis prophylaxis. Celecoxib 100 mg twice daily was started based on prior studies indicating that NSAIDs may reduce HO formation after hip surgery [11].

Surgery was pursued to stabilize the fracture, reduce pain, and preserve potential mobility. The surgical approach was designed to minimize soft tissue trauma but provide adequate access to safely stabilize the fracture in a way that would allow immediate weight-bearing. The patient underwent intramedullary nailing of the femur shaft with screw placement at the distal femur 9 days after the fall, after transfer to University of California, San Francisco (UCSF). Asleep nasal fiberoptic intubation with a small tube was carefully performed after induction of general anesthesia. This approach was used to minimize trauma which could trigger an obstructing neck flare leading to cervical spine fusions, ankyloses of the temporomandibular joint, or airway compromise. Methylprednisolone 50 mg IV was administered at the time of surgery to decrease the risk of airway edema and 2 g cefazolin was administered prior to incision for antibiotic prophylaxis. Bony prominences were carefully padded to prevent pressure-induced flare-ups. The jaw was also propped closed with foam tucked under the chin to prevent stretching and injury to the masseter muscles. After surgery, he was started on prednisone 50 mg twice daily for 1 day and then tapered by 10 mg/day until completion.

Manual traction was employed to position the hip to restore length; however, as varus was still noted, a pin was placed through the planned skin incision for nail insertion from the top of the greater trochanter across the base of the femoral neck fracture into the calcar in order to anatomically reduce the displaced fracture. Afterward, a threaded tip guidewire was placed to guide trochanteric entry of the nail. A 4 cm incision was made for passage of the trocar and entry reamer and a ball-tipped guidewire was placed across the fracture and to the level of the superior pole of the patella within the medullary cavity of the femur. This was followed by passage of serial reamers starting at 10 mm and advancing in 1 mm increments up until endosteal chatter was obtained at 15 mm. The final reaming diameter was 15.5 mm. A 13 mm trochanteric entry cephalomedullary nail was attached to the insertion jig and inserted (Trochanteric Fixation Nail, DePuy Synthes, West Chester, Pennsylvania). Using fluoroscopic imaging, the nail was positioned to allow for passage of a spiral blade in a central position within the femoral head and neck. A guidewire was passed using imaging guidance into the subchondral bone of the femoral head and the spiral blade was inserted over the guidewire through the proximal aspect of the nail via a second lateral proximal thigh incision. A single distal interlocking screw was placed using freehand technique via a 2 cm incision on the distal lateral thigh. The position was confirmed on fluoroscopic imaging. Care was taken to irrigate any bone from the soft tissues at the nail, spiral blade, and distal interlocking screw site in order to minimize the risk of HO.

After surgery, flare-up based palovarotene dosing was started at 20 mg daily for 4 weeks and then decreased to 10 mg/day for an additional 8 weeks per the study protocol. Due to repeated flare-ups he was not able to decrease his palovarotene to the 5 mg/day chronic dosing. Post-operative celecoxib was continued.

Radiologic and clinical assessments did not show any delay in fracture healing. Over the subsequent 2 months, the patient showed progressive clinical loss in the range of motion of the left hip and required the use of a cane for ambulation. A review of X-ray images at the time of the fracture showed no evidence of HO at the left hip (Fig. 2a). Radiologically, HO and a callus were evident around the distal screw by the left knee after 4 months (Fig. 2b). In addition, HO at the left hip was evident by 9 weeks and fully matured by 14 weeks (Fig. 2c). Brooker class D HO was clearly present on imaging at 9 months after surgery and matured at 12 months after surgery [12] (Fig. 2d-e).

Nine months after the left femoral fracture, the patient was on palovarotene 10 mg/day after a separate series of flare-ups. Four days after tapering to palovarotene 10 mg/day dose, he had an intercurrent flare in his chest that was treated with palovarotene 20 mg/day. The patient had a second fall 4 days later, where he lost his balance and fell backwards resulting in a subdural hematoma, left parietal bone fracture, and a displaced right sub-trochanteric femoral fracture. Palovarotene was continued at 20 mg/day per a study modification allowing for prophylactic treatment of a physical injury, and the patient received prednisone 100 mg/day for 4 days followed by a taper by 20 mg every 2 days, starting on the day of the injury.

The risks and benefits of non-operative management versus surgery were discussed again with the patient. Adequate pain control was difficult during the non-operative treatment. After transfer to UCSF and stabilization of his other injuries, he underwent intramedullary nailing of the right hip 10 days after sustaining the fracture.

Despite straightforward asleep nasal fiberoptic intubation during the prior surgery, repeat attempt at nasal intubation for this procedure was difficult, requiring multiple attempts and eventual oral intubation with a video laryngoscope. Once his airway was stabilized, the identical long cephalomedullary nail was used. Antibiotic prophylaxis with 2 g cefazolin at the time of incision was again used. However, on the right side we avoided the use of a distal interlocking screw as its use is not required for successful fixation and stabilization of such injuries and may have contributed to the HO around the knee after his prior surgery. The intertrochanteric fracture was noted to be in some degree of varus and the femur minimally shortened, but in order to minimize soft tissue injury, an open reduction was not performed. No traction pins or additional bony manipulation was performed. Under fluoroscopic imaging, the appropriate incision was mapped out proximal to the greater trochanter and in line with the femur in the sagittal plane. A threaded tip guidewire was once again positioned to direct nail entry using fluoroscopic imaging. An entry reamer was inserted with as little trauma as possible through the soft tissues and passed into the proximal femur. This was followed by placement of a ball-tipped guidewire into the distal aspect of the femur. A 15.5 mm reamer was attached to the reaming drive and passed in a single pass without any evidence of compromise to the surrounding endosteal surfaces of the femur. A 13 mm trochanteric fixation nail was inserted in an antegrade fashion. A lateral cortical drill and reamer was used to open up the canal for passage of the spiral blade. Fluoroscopy was used to confirm restoration of alignment and correct positioning of all implant components. He remained intubated after the procedure and was extubated without incident on post-operative day 1. Fortunately, he suffered no significant complications from a longer duration of intubation or HO formation in the temporomandibular joint or surrounding tissues.

**Fig. 3:**
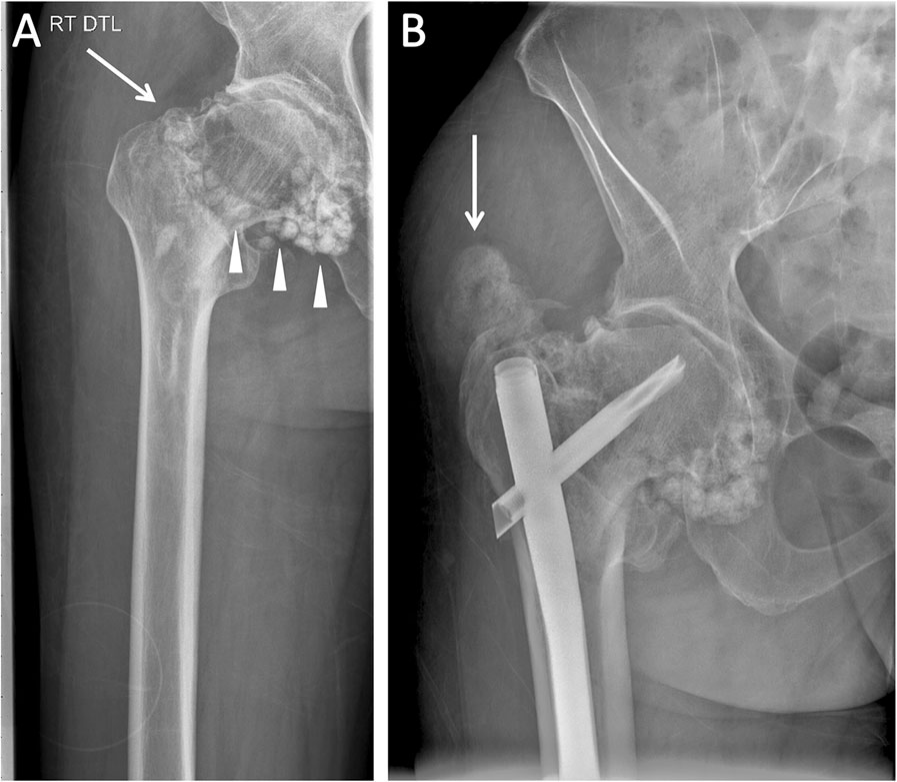
X-Ray imaging of the right hip: (**a**) Intertrochanteric fracture noted by arrow. There is longstanding osteochondromatosis at the right femoral head [arrowheads]. (**b**) X-ray of the femur 13 weeks after surgery show post-surgical hardware and demonstrate Grade B HO at the left femoral head [arrow]

Fracture healing was not delayed based on clinical imaging; however, the patient showed continued loss of mobility, requiring the use of a walker and wheelchair. Again, the patient showed multiple and prolonged FOP flare-up activity, resulting in a significant increase in the number of flares/year. The right hip showed new development of Brooker class B HO at the greater trochanter, near the insertion site of the intramedullary rod, by 3 months after surgery (Fig. 3).

## Discussion and conclusions

Here, we describe the first case using the RAR-γ agonist, palovarotene, in a patient with FOP undergoing hip fracture repair surgery. It has been shown that chondrogenesis requires transcriptional repression of RARs and that retinoic acid receptor activation blocks chondrogenesis. Activation of RAR-γ, expressed in chondrogenic cells and chondrocytes, can selectively inhibit HO [7, 13, 14]. Initial studies by Shimono et al. with RAR agonists in mouse mesenchymal stem cells showed decreased phosphorylation of SMAD1, SMAD5, and SMAD8 and overall SMAD levels, suggesting a reduction in BMP signaling [7]. This led to studies of the use of palovarotene in trauma-induced mouse models of FOP. Chakkalakal et al. demonstrated a significant reduction in HO formation and fibroproliferative responses in knock-in Acvr1^R206H^ mice when palovarotene was administered after cardiotoxin induced injury [6]. Joint and limb mobility was preserved in these palovarotene-treated mice.

Phase 2 (NCT02279095) and 3 clinical trials (NCT03312634) to test palovarotene in patients with FOP are currently on-going. A prior study of palovarotene for treatment of emphysema showed that the drug was well tolerated with skin, gastrointestinal, eye, and respiratory mucocutaneous side effects being most common at the dosing regimen used in that patient population [15].

Despite the known link between retinoids and skin adverse events, the patient described here had normal wound healing and showed no unexpected or major skin adverse events. The patient received topical prophylaxis with Aquaphor barrier ointment, triamcinolone 0.1% for retinoid skin reactions such as rash and dry skin, and artificial tears, for managing the most common potential mucocutaneous side effects. Notably, the patient showed no delayed healing of his skin wound or tissue after either surgery. Thus, the high-dose retinoid was well tolerated by this patient in the setting of two surgeries. Palovarotene did not have a significant or negative impact on clinical fracture healing or osteointegration with the repair hardware.

The bilateral fragility fractures were likely multifactorial in origin. The patient had a history of prolonged and high dose prednisone throughout childhood and adulthood. CT bone densitometry performed after the first fracture showed a lumbar spine T-score 3.8 standard deviations below the reference population, consistent with a diagnosis of severe osteoporosis. This likely arose from both decreased weight bearing as well as steroid-induced osteoporosis despite having received IV zoledronate on a regular basis. Osteoporosis may be a major clinical feature of advanced FOP since severe bone thinning is clearly evident in the two FOP skeletons housed in the Mutter Museum. Shifts in calcium from the native skeleton to inappropriate sites have also been reported in patients with severe atherosclerosis [16, 17], suggesting that shifts in calcium stores may be a contributor to FOP related degeneration of the native skeleton. In addition, the frequent and long-term use of bisphosphonates in this patient raised the possibility that the patient was predisposed to atypical-like femur fractures. In our patient, he reported no prodrome symptoms, and the fractures occurred in the setting of clear trauma.

In general, patients with FOP should avoid surgery unless absolutely necessary because of the high risks of complications from intubation, the surgical procedure, and triggering of FOP flares. Although the patient was successfully intubated, the second intubation was extremely difficult and emphasizes the high risks of surgery on FOP patients. It is unclear whether this was due to the presence of skull fractures, residual facial soft tissue swelling, HO from the prior intubation, or some combination thereof. Fortunately, he suffered no significant complications from a longer duration of intubation or HO formation in the temporomandibular joint or surrounding tissues as a result. In addition, HO formed in areas near surgical interventions such as the hardware insertion tract through the soft tissues and around the locking screw above the left knee. The prolific formation of HO after surgeries in FOP has been described before [18].

The formation of HO at the hardware insertion sites raises the concern that the reaming process may have contributed to HO formation. The physiologic effects of canal reaming can disrupt cortical blood flow but appears to be offset by increased circulation to the periosteum and surrounding skeletal muscle with subsequent revascularization that ultimately promotes healing [19, 20]. Investigations into reaming debris have showed elevated levels of FGF, PDGF, IGF-1, TGF-B and BMP-2 [21]. While these factors might usual induce recruitment and migration of progenitor cells into the fracture, stimulating osteogenesis and angiogenesis, in a patient with FOP this may lead to further activation of the ACVR1/Smad cascade. This stimulation could, in theory, contribute to HO formation in patients with FOP; however, as there was a concern for nonunion, stabilization of the fracture sites was the highest priority which led to the decision to proceed with reaming. To minimize trauma, biopsies were not obtained from the fracture sites.

Notably, the patient developed a significant increase in flare activity after each fall. The occurrence of multiple flares after the trauma likely resulted in further HO formation, and makes it difficult to assess whether palovarotene reduced HO formation since the inflammatory stimuli were persistently present. In addition, the second fall involved multiple injuries consistent with poly-trauma, which in and of itself is associated with increased risks of heterotopic ossification [22] such as in survivors of high-energy trauma. Although it is difficult to compare the left and right fracture events, there was significantly less heterotopic ossification on the right side (second fracture) as compared to the left side (which involved more surgical manipulation). It is worth noting that minor clinically evident HO formation was eventually detectable after the extensive manipulation of the temporomandibular joint and upper airways during the difficult intubation for the second surgery. Whether administration of palovarotene played a role in avoidance of facial and airway HO formation is unknown. These observations, as well as the finding of multiple subsequent “after-shock” flares after the primary injury, further reinforces the importance of judicious surgical management for patients with FOP and the likely correlation between the degree of injury and the severity of the subsequent HO. These observations also suggest that future therapeutic strategies to control the inflammatory response after the inciting injury may be useful considerations for breaking the prolonged cycles of flares reported by FOP patients, and that these cycles may increase the risk of heterotopic bone formation.

Though this case involves a patient with a genetic form of HO, testing of RAR-γ agonists in non-genetic forms of HO may still be beneficial. Non-genetic HO has been demonstrated to occur in adults, typically in their second and third decades, after a history of trauma or repetitive mechanical stress and can range from being clinically unnoticeable to resulting in chronic pain and high morbidity [23]. HO has been noted particularly in the setting of combat related injury such as post-blast injury or extremity amputation, where the prevalence of HO formation can approach 65% of patients [22]. Orthopedic surgery, including hip arthroplasty, fracture, traumatic brain or spinal cord injury, and severe burns are risk factors in the development of heterotopic ossification. The pathogenesis is thought to be similar to HO development in FOP, where inflammation leads to recruitment of progenitor cells and ultimately endochondral or intramembranous ossification but there are multiple pathways involved [24]. Studies in non-genetic HO models are limited. A selective RAR-α agonist was tested in an rhBMP-2 mouse model and reduced heterotopic cartilage, bone and expression of chondrogenic genes [25]. A study of palovarotene in a blast-related traumatic injury mouse model decreased HO formation by 50–60% and decreased ectopic chondrogenesis, osteogenesis and angiogenesis at the injury site [26]. Palovarotene showed slight inhibitory effects on wound healing, but this was not statistically significant. Thus, RAR-γ agonism may still be beneficial for non-genetic forms of HO.

Current treatment for FOP [5] is largely limited to avoidance of trauma and supportive measures such as glucocorticoids for flare management, COX-2 inhibitors, non-steroidal anti-inflammatory, bisphosphonates, mast cell inhibitors, and potentially imatinib [27] or IL-1 inhibitors [28]. Surgery should still be avoided in FOP patients whenever possible; however, if surgical intervention is needed, the most minimally invasive approach should be used.

Our patient demonstrates that careful treatment of a patient with FOP undergoing surgery with a retinoic acid receptor agonist such as palovarotene can be well tolerated with minimal side effects. However, major trauma may increase FOP flare activity that would complicate end-point assessments for clinical trials of medications for surgical treatment in FOP. Thus, fracture healing, flare activity, and HO formation are key endpoints that should be assessed in clinical trials involving surgical outcomes in novel FOP therapies [29]. Finally, it remains to be seen if palovarotene or other blockers of chondrogenic ossification may be useful for the treatment of non-genetic HO in more common conditions such as hip arthroplasty.

## Data Availability

The data that support the findings of this study are available from the corresponding author upon reasonable request.

## Abbreviations

FOP: Fibrodysplasia ossificans progressiva
HO: Heterotopic ossification
NSAIDs: Non-steroidal anti-inflammatory agents
RAR: Retinoic acid receptor
